# Novel Tools for Diagnosis and Monitoring of AML

**DOI:** 10.3390/curroncol30060395

**Published:** 2023-05-23

**Authors:** Francesca Guijarro, Marta Garrote, Neus Villamor, Dolors Colomer, Jordi Esteve, Mónica López-Guerra

**Affiliations:** 1 Hematopathology Section, Pathology Department, Hospital Clinic Barcelona, 08036 Barcelona, Spain; fguijarro@clinic.cat (F.G.); garrote@clinic.cat (M.G.); villamor@clinic.cat (N.V.); dcolomer@clinic.cat (D.C.); 2 Institut d’Investigacions Biomèdiques August Pi I Sunyer (IDIBAPS), 08036 Barcelona, Spain; jesteve@clinic.cat; 3 Centro de Investigación Biomédica en Red de Cáncer (CIBERONC), 28029 Madrid, Spain; 4 Hematology Department, Hospital Clinic Barcelona, 08036 Barcelona, Spain

**Keywords:** AML, diagnosis, MRD monitoring, flow cytometry, NGS, digital PCR

## Abstract

In recent years, major advances in the understanding of acute myeloid leukemia (AML) pathogenesis, together with technological progress, have led us into a new era in the diagnosis and follow-up of patients with AML. A combination of immunophenotyping, cytogenetic and molecular studies are required for AML diagnosis, including the use of next-generation sequencing (NGS) gene panels to screen all genetic alterations with diagnostic, prognostic and/or therapeutic value. Regarding AML monitoring, multiparametric flow cytometry and quantitative PCR/RT-PCR are currently the most implemented methodologies for measurable residual disease (MRD) evaluation. Given the limitations of these techniques, there is an urgent need to incorporate new tools for MRD monitoring, such as NGS and digital PCR. This review aims to provide an overview of the different technologies used for AML diagnosis and MRD monitoring and to highlight the limitations and challenges of current versus emerging tools.

## 1. Introduction

Acute myeloid leukemia (AML) is characterized by a high biological heterogeneity both at diagnosis and during disease evolution. In this context, an exhaustive characterization of immunophenotypic and molecular profiles is required to guide the clinical management of AML patients. In recent years, growing evidence has shown the prominent role of cytogenetic and mutational data in AML and, accordingly, the recently updated World Health Organization (WHO) [1] and International Consensus Classification (ICC) [2] classifications of myeloid neoplasms as well as the European LeukemiaNet (ELN) recommendations [3] have incorporated genetics into their diagnostic, prognostic and therapeutic algorithms. In this scenario, a combination of immunophenotyping and genetic studies are required for AML diagnosis and follow-up. Measurable residual disease (MRD) has strong prognostic and predictive value in AML, and its accurate monitoring is crucial for disease management. The plethora of aberrant phenotypic and molecular characteristics of AML cells represents a chance to track this disease but, at the same time, constitutes a major challenge for hematopathology laboratories. In this review, we discuss the most relevant technologies based on flow cytometry and molecular biology used for AML diagnosis and MRD monitoring in the context of recently updated diagnostic and prognostic guidelines.

## 2. Diagnosis

### 2.1. Flow Cytometry for Lineage Assessment

Multiparametric flow cytometry (MFC) consists of the recognition of cells based on antigen detection through a combination of fluorochrome-labeled monoclonal antibodies, which allows phenotypic characterization and the quantification of a specific cell population.

**Figure 1:**
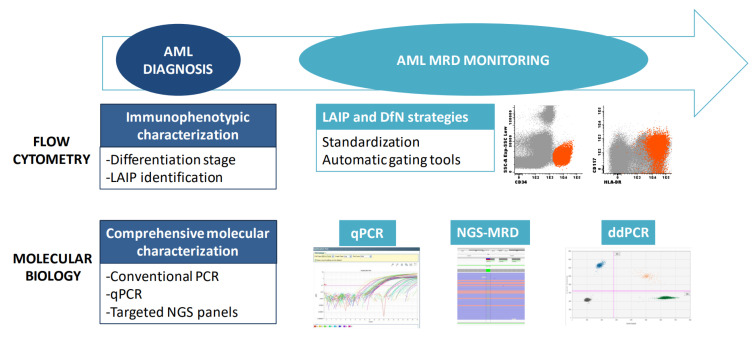
Tools for diagnosis and monitoring of AML. A combination of immunophenotyping and molecular studies is required for AML diagnosis and MRD monitoring. MRD: Measurable residual disease; LAIP: Leukemia-associated immunophenotype; DfN: Different from normal; qPCR: quantitative PCR; ddPCR: Droplet digital PCR.

When acute leukemia is suspected, morphologic and immunophenotypic examinations of the bone marrow and peripheral blood are the quickest techniques to confirm the diagnosis (Figure 1). The latter is also essential when assigning the leukemic lineage: a crucial step that determines further diagnostic tests and therapeutic strategies. Lineage-assigning antigens are shown in Table 1. Myeloperoxidase (MPO) expression is the hallmark of myeloid commitment, though it is not always present. AML with minimal differentiation is defined by an absence of lineage-assigning antigens in combination with the expression of at least two other myeloid-related antigens (e.g., CD13, CD33, CD117). Monocytic AML often loses MPO expression and is characterized by specific markers such as CD11c, CD14, CD64 and lysozyme.

AML differentiation, maturation stage and the aberrant co-expression of lymphoid markers can be only addressed with a broad panel of antibodies. Morphologic and immunophenotypic criteria for cases without recurrent genetic abnormalities have been updated in the latest WHO classification [1]. Even when this information lacks its independent prognostic impact, it often correlates with particular genetic lesions. For example, acute promyelocytic leukemia (APL) exhibits a characteristic immunophenotype (CD34−, HLA-DR−, CD117+, CD15−) that can prompt *PML::RARA* gene fusion evaluation and consequent management until the molecular result is available. Another example is the association between the co-expression of CD25, CD123 and CD99 on myeloid blast and *FLT3* mutations [4].

The Euroflow Consortium has developed, through serial testing, an acute leukemia orientation tube (ALOT), which includes most of the lineage-defining markers, and a panel for AML that covers the different myeloid lineages and most frequent lymphoid aberrancies [5]. Moreover, the standardization of MFC and the building of reference databases with normal and acute leukemia samples allows for automated gating identification of leukocyte subpopulations and classification of blast cells [6,7].

The use of a comprehensive panel of antibodies also allows for the recognition of leukemia-associated immunophenotype (LAIP) patterns that can be of interest during follow-up. Finally, new therapies targeting the surface antigens of myeloid blasts are under development. In the future, flow cytometry could be useful when identifying potential candidates for treatment against not-universally expressed antigens, as could be chimeric antigen receptor therapy against CD123 [8].

### 2.2. Molecular Biology

Over the last decade, genomic studies based on Next-Generation Sequencing (NGS) have dissected the molecular profile of AML, with the description of new mutations, copy number variations and recurrent fusion genes [9,10]. The growth in molecular knowledge has prompted the update of both diagnostic and management recommendations for AML patients. The recently updated WHO, ICC and ELN classifications have undertaken a major role in genetic data and have been integrated into diagnostic and prognostic algorithms (Table 2) [1,2,3]. In this context, genetic analyses currently mandatory in the evaluation of AML are conventional cytogenetics together with the screening of fusion genes and of gene mutations, whose number has significantly increased in comparison with prior classifications (Figure 1). Recommendations for molecular assessments in AML include the following genes: *FLT3* (both internal tandem duplications, ITD, and tyrosine kinase domain, TKD, mutations), *IDH1* and *IDH2* as therapeutic targets, and *NPM1*, *CEBPA*, *DDX41*, *TP53*, *ASXL1*, *BCOR*, *EZH2*, *RUNX1*, *SF3B1*, *SRSF2*, *STAG2*, *U2AF1* and *ZRSR2* as diagnostic/prognostic disease markers. Furthermore, the screening of the most prevalent gene rearrangements include *PML::RARA*, *RUNX1::RUNX1T1*, *CBFB::MYH11*, *KMT2A* rearrangements, *BCR::ABL1* and others and should be performed when rapid information is needed for the clinical management of a patient, or if the morphology and/or immunophenotype are highly suggestive of the presence of a particular fusion gene [3].

In this scenario, the screening of single genes is progressively being replaced by targeted NGS panels, which, in addition, could offer a combined solution for the parallel analysis of DNA for mutations and RNA for fusions in the same assay. However, the main limitation of NGS technology is that it requires batching to be cost-effective, making it unrealistic for most diagnostic laboratories to report NGS results within a week. In this context, conventional PCR-based techniques are still being used for the screening of relevant markers that require a rapid turn-around time (3–5 days), including *NPM1*, *FLT3*, *IDH1/2*, and fusion genes. In particular, *FLT3*-ITD evaluation is recommended to be performed by capillary electrophoresis [11] because it cannot be performed robustly by NGS.

Among the most relevant molecular changes included in the new classifications is the description of some new AML types with fusion genes such as *DEK::NUP214*, *BCR::ABL1* or rearrangements involving *KMT2A*, *MECOM* and *NUP98* (Table 2). Another remarkable change refers to the recognition of particular clinicobiological entities associated with adverse risk, AML with a *TP53* mutation and the renamed AML with myelodysplasia-related gene mutations (ICC 2022)/AML myelodysplasia-related (WHO 2022), which harbor specific cytogenetic and molecular alterations beyond those previously considered *ASXL1* and *RUNX1* (Table 2). Regarding AML with *CEBPA* mutations, it remains a favorable prognostic entity, but a biallelic mutation is not required, according to several studies published; a single in-frame mutation in the basic leucine zipper (bZIP) region of *CEBPA* is sufficient for diagnosis [3,12,13]. At the prognostic level, all AML patients with *FLT3*-ITD are classified in the intermediate risk group irrespective of the *FLT3*-ITD ratio and the presence of the *NPM1* mutation. Furthermore, the presence of adverse-risk cytogenetic aberrations in *NPM1*-mutated AML is now also indicative of high risk [3].

The study of an additional set of genes, including *ANKRD26*, *BCORL1*, *BRAF*, *CBL*, *CSF3R*, *DNMT3A*, *ETV6*, *GATA2*, *JAK2*, *KIT*, *KRAS*, *NRAS*, *NF1*, *PHF6*, *PPM1D*, *PTPN11*, *RAD21*, *SETBP1*, *TET2* and *WT1*, is recommended at AML diagnosis, and some of them can also be used for MRD monitoring [3,14]. When AML with germline predisposition is suspected, mutational studies should be complemented with an extended NGS panel covering known predisposing genes together with genetic counseling. Both ICC and WHO updated classifications include specific categories of myeloid neoplasms with a germline predisposition characterized by the presence of germline inherited mutations and specific clinical features [1,2], but the discussion of these entities is beyond the scope of this review.

## 3. Monitoring

Though classical AML response criteria rely on morphological blast count, both MFC and molecular techniques offer higher sensitivity and specificity for the identification of malignant cells after therapy. Since 2017, ELN response criteria have included a complete response without MRD, recognizing the value of a deeper remission status. MRD detection is highly predictive of relapse [15,16] and allows for early intervention (MRD-directed therapy). Retrospective studies suggest pre-emptive treatment before overt morphological relapse and can improve the results of salvage treatment [17,18]: a hypothesis that is currently being addressed by prospective clinical trials (CT) [19]. In the allogeneic transplantation setting, the presence of MRD before this procedure also increases the risk of relapse [20] and could guide toward more intensive conditioning regimens [21]. Finally, MRD has been proposed as a surrogate endpoint for CT, which could accelerate drug approval.

Nonetheless, being aware of the sensitivity and limitations of each particular methodology is essential for the correct interpretation of MRD results. Thus, MRD negativity does not necessarily mean leukemia eradication but rather indicates a leukemic load below the limit of detection for each particular assay. Furthermore, MRD reliability is related to sample quality, as sensitivity is directly dependent on the number of processed cells, and specificity may be affected by sample viability. To guarantee as many viable cells as possible, it is recommended that BM aspiration is first pulled using the first 48 h for MRD studies while prioritizing MFC [22].

### 3.1. Flow Cytometry for MRD Detection in AML

Indeed, many studies have proven that the identification of leukemic cells by MFC after induction therapy or during consolidation treatment is a strong indicator of a higher relapse risk with an impact on overall survival [23,24,25,26]. There is also evidence of the MRD prognostic value in a low-intensity therapy setting [27,28]. The ELN MRD Working Party proposes an MFC-MRD assessment based on the detection of immunophenotypic anomalies of leukemic cells following LAIP [29] and is different from normal (DfN) [30] strategies. The LAIP strategy is based on identifying abnormal antigen patterns that are detected at diagnosis. The DfN approach is applicable to patients for whom the diagnostic phenotype is unknown and does not rely on its stability over time.

MFC is the most commonly used technology for MRD detection [22] because of its widespread use in most hematopathology laboratories and the fact that it is applicable to 85–90% of patients with AML [3] with a limited cost (Figure 1). Another advantage of MFC is that it is able to detect signs of hemodilution through the examination of other cell subsets [31]. Moreover, forward and side scatter properties hint at the overall sample quality, which can be more accurately assessed using a viability dye [31]. Modern cytometers can process several millions of cells in a few minutes, analyzing at least eight markers at a time. Assuming a tube composition is able to distinguish the aberrant phenotype from background noise, theoretical sensitivity lies between 10^−3^ and 10^−5^. However, the clinically validated threshold proposed by ELN recommendations is only 0.1%, which is lower than the sensitivity cut-off of many molecular techniques due to technical limitations and interpretation complexity. One of the main caveats of MFC is its low reproducibility, which is caused by differences in equipment, cytometer set-up, sample processing protocols and reagents across institutions. Even though there is a set of markers that are considered basic for AML MRD detection (Table 1), tube combination is highly variable and can greatly determine the gating strategy. In addition, AML is phenotypically highly heterogeneous, both in terms of the wide range of possible aberrancies as of intra-tumor complexity with different subclones, which may exhibit particular immunophenotypes. Moreover, LAIP shifts are not rare at relapse, and certain aberrancies can be found in healthy and regenerating bone marrow at low frequencies, which compels specificity thresholds for every specific assay [32]. Expertise is required for every examiner to overcome interpretation complexity [22]. Recently, some publications have addressed the standardization of MRD analysis by MFC in order to improve the inter-rater reliability between investigators [31,33]. Furthermore, automated analysis tools are being developed to achieve unbiased results [34,35,36]. With standardized assays, deeper sensitivity thresholds will probably be accepted for clinical use in the future.

The study of the leukemia stem cell (LSC) compartment is still considered investigational, though its prognostic value has been demonstrated in prospective studies [37,38] and, thus, may also be recommended for clinical routine analysis in the near future.

Spectral flow cytometry allows for the simultaneous analysis of more than 20 markers, which can greatly increase its capability to identify immunophenotypic aberrancies and increase the number of analyzed cells when a sample is scarce [39]. Nevertheless, this methodology is still investigational and far from being implemented in most laboratories.

### 3.2. Molecular Monitoring

The advent of precision medicine, together with technological advances, has led to a new era in molecular AML monitoring, with the challenge of choosing the most appropriate technique and the best molecular marker for each patient. At a technical level, molecular MRD should be assessed by methodologies with a limit of detection of at least 10^−3^. To achieve this value, the technologies currently recommended by the ELN in AML are real-time quantitative PCR (qPCR), digital PCR (dPCR) and NGS (Figure 1), taking into account that dPCR and NGS are still exploratory and need to be integrated with MFC-MRD data [22].

#### 3.2.1. qPCR

Real-time quantitative PCR (qPCR) was the first molecular methodology to be established for MRD leukemia monitoring because of its capability to accurately quantify the mutational load of several genetic aberrations with high sensitivity (10^−5^–10^−6^) [40]. qPCR allows for the real-time measurement of PCR amplification products by means of fluorescent assays that are specifically designed for each alteration, including fusion genes as well as gene mutations. For many years and still today, qPCR has been the gold-standard technique for monitoring AML patients harboring the fusion genes *PML::RARA* in APL and *RUNX1::RUNX1T1* and *CBFB::MYH11* in core-binding factor (CBF) leukemias [41,42,43]. The methodology and interpretation of qPCR for these fusion genes have been standardized by Europe Against Cancer (EAC) Consortium [40,44], with a wide application in routine patients’ clinical management because of their simplicity, relatively low cost and high sensitivity. In addition to APL and CBF leukemias, the clinical value of MRD monitoring has been also well established and is strongly recommended in AML patients with *NPM1* mutations [45,46]. The high gene expression of these fusion genes and *NPM1* mutations allows for the analysis of RNA as a preferred starting material with excellent sensitivity and facilitates the study of fusion genes without requiring the particular location of each intronic breakpoint [47]. Therefore, in patients with these alterations, the molecular monitoring of aberrant transcripts should be performed at the time points at which MRD is considered clinically relevant, with a high predictive value for relapse, as defined in the latest ELN recommendations [22]. Although the analysis in the peripheral blood is currently accepted at some time points, it should be noted that MRD assessment in bone marrow has higher sensitivity by an order of magnitude compared with blood [48].

qPCR can also be used to monitor other MRD targets through the design of particular assays that are specific for each type of genetic alteration. Thus, qPCR is also applicable in the analysis of other recurrent fusion genes, such as *KMT2A* rearrangements or the *DEK::NUP214* fusion resulting from t(6;9)(p22.3;q34.1), as well as atypical *NPM1* mutations and *KMT2A* partial tandem duplications [46,49,50]. All these targets are clinically valuable MRD markers; however, the qPCR-based methodology used was not standardized given the lack of studies with large series of patients. Despite its high sensitivity and robustness, the main drawback of qPCR is its limited applicability. About 50% of AML patients do not carry *NPM1* mutations or recurrent fusion genes, and in this context, there is an urgent need to introduce novel molecular MRD techniques in order to monitor a broader range of AML patients.

#### 3.2.2. Digital PCR

Droplet digital PCR (ddPCR) is the most extensively used dPCR technology and has emerged as an attractive method to monitor MRD while overcoming some limitations of conventional techniques. ddPCR can be more accurate than qPCR, particularly in disease quantification at a very low level, as each sample is initially fractionated and the final analysis is based on thousands of individual measurements, obtaining an absolute quantification of the amplified target of interest without the need for a standard curve [51]. This provides ddPCR with the ability to repeatedly monitor patients’ MRD with high sensitivity. The limit of detection in this technique depends on several aspects, including the specific design assay or the number of replicates per sample, but in any case, should be at least 10^−3^ according to ELN recommendations. In recent years, several studies have shown the potential of ddPCR to analyze MRD in AML, both in monitoring somatic mutations and fusion genes [52]. The feasibility of ddPCR to track MRD in AML has been explored in different studies, in particular, for *NPM1*-mutated patients, with the advantage of atypical mutation screening through the design of specific assays [53]. Based on the published data, the last ELN update already included dPCR, together with qPCR, as one of the recommended technologies for the molecular MRD assessment in mutant *NPM1*, CBF AML or APL. Regarding the monitoring of other recurrent mutations, some studies have shown the potential of ddPCR for predicting AML relapse, including *IDH1/2* [46,54]. However, the value of some of these mutations as potential MRD markers should be validated in additional studies with larger cohorts of patients.

For the MRD assessment of rare mutations, ddPCR could be useful due to its versatility when designing alteration-specific assays, but at the same time, this could also be a pitfall due to the lack of standardized assays. For this reason, to date, the potential of ddPCR disease tracking in cases with specific gene alterations is still under investigation, and its MRD results must be interpreted together with MFC data.

#### 3.2.3. NGS

As discussed above, the use of targeted NGS panels is one of the most extended approaches when accurately diagnosing and stratifying risks in AML patients. In addition to initial diagnosis and relapse, NGS has recently become a potentially useful tool for monitoring MRD in AML due to the development of error-corrected NGS, which increases sensitivity through the use of random barcodes or unique molecule identifiers [55]. These strategies allow for the identification and removal of artifacts introduced by PCR amplification during library preparation, leading to the reliable and accurate tracking of genetic targets with a limit of detection of at least 10^−3^. Due to its intrinsic characteristics, NGS-MRD provides a potential solution to some limitations of the conventional molecular techniques due to its capability to monitor in the same run of sequencing specific and various gene alterations in virtually all AML patients. Thus, several studies have shown that MRD monitoring by NGS has a predictive value for AML relapse, as well as prognostic value, together with an excellent correlation with treatment efficacy [16,56,57]. However, despite being promising, these strategies require highly specialized bioinformatic tools that are not yet available in most routine laboratories. Moreover, some technical issues need to be solved and standardized before entering into clinical practice. In this scenario, there needs to be an increasing cooperative effort to harmonize NGS-MRD protocols. Partly due to this lack of standardization, the best strategy is to combine NGS with MFC for MRD assessment [58].

#### 3.2.4. Selection of MRD Molecular Markers

Although all pathogenic/likely pathogenic variants detected at AML diagnosis could be considered potential MRD markers, some limitations should be taken into account (Table 3). First, confirmed or suspected germline mutations with a variant allele frequency higher than 30% in *ANKRD26*, *CEBPA*, *DDX41*, *ETV6*, *GATA2*, *RUNX1* and *TP53* genes, should be excluded as they are not informative for disease monitoring [22]. Among the somatically acquired mutations, it is crucial to distinguish those that are specific from leukemic cells with the potential to relapse and those related to pre-leukemic clonal hematopoiesis (CH). In this context, mutations known as DTA (affecting *DNMT3A*, *TET2* or *ASXL1* genes) often persist in complete remission (CR) without being associated with a higher risk of relapse and should be excluded from MRD tracking [16,59,60]. Regarding other less frequently mutated genes related to CH, including non-DTA, such as *SRSF2*, *IDH1* or *IDH2*, preliminary studies seem to indicate that their detection at CR is not associated with AML relapse [61], although larger studies are needed to confirm this result.

On the contrary, late event mutations in genes with a transformative leukemic capacity, such as *NPM1*, *FLT3*, *RUNX1* or *RAS*, are considered good MRD markers, as their detection in CR can be associated with an increased risk of relapse, representing residual leukemic cells when detected [59]. Nonetheless, mutations in signaling kinase pathways (*FLT3*, *RAS*, *KIT*) are usually subclonal and are not always present at relapse; therefore, it is highly recommended to conduct an analysis in combination with additional molecular markers. In patients treated with targeted agents (FLT3, IDH1/2 inhibitors), the molecular monitoring of targeted markers is recommended in combination with others [22]. In fact, the analysis of additional markers may also be applied even in *NPM1* mutated AML patients, given the not negligible frequency of wild-type *NPM1* when relapsed among AML patients with an *NPM1* mutation [46,62]. All this accumulated evidence may favor the use of a multigene MRD panel as a strategy to target several mutations per patient, excluding DTA and genes related to germline predisposition.

## 4. Conclusions

Over the last decade, the growing knowledge of the pathogenesis of AML, together with new technological advances and the success of targeted therapies, have led to a new era in the diagnosis and follow-up of AML. In this context, guidelines for disease classification, risk stratification and MRD monitoring have been recently updated, prompting routine laboratories to incorporate new diagnostic and monitoring tools in order to optimally characterize and track leukemic cells. Due to the remarkable AML biological heterogeneity, both flow cytometry-based as well as molecular procedures require specialized expertise and, in this context, harmonization, which is of critical importance. Cooperative efforts are underway, which is particularly challenging for MRD studies, given their strong relevance in AML clinical practice. Automated unsupervised analysis strategies may improve the standardization of MFC-MRD assays and overcome the subjectivity associated with flow cytometry. Emerging technologies, such as NGS-MRD and dPCR, should be incorporated into the arsenal of MRD techniques, taking into account that, although promising, they are still complementary and need to be integrated with other laboratory data.

## Figures and Tables

**Table 1:** Flow cytometry diagnostic and MRD markers.

Diagnostic Markers	
Lineage assigning antigens	
Myeloperoxidase	Myeloid lineage
CD11c, CD14, CD64, lysozyme	Myeloid lineage with monocytic differentiation
CD19	B-lineage. Requires also at least one (CD19 strong) or two (CD19 weak) from CD22, CD10 and CD79a
CD3 (surface or cytoplasmic)	T-lineage
Myeloid differentiation-associated antigens	
CD13, CD33, CD11b, CD15, CD64	Myeloid
CD14, CD36, CD64, CD4, CD11c	Monocytic
CD41, CD42b, CD61, CD36	Megakaryocytic
CD235a, CD71 strong, CD105, CD36	Erythroid
CD203c, CD123	Basophil
CD123, CD4, HLA-DR strong, CD303, CD304	Dendritic
CD117 strong	Mastocytic
**MRD markers**	
Basic markers	
CD34, CD117, HLA-DR, CD45, CD13, CD33	Myeloid precursor identification
CD7, CD56	Lymphoid antigen aberrancies
Other useful markers	
CD64, CD14, CD11b, CD4	Monocytic
CD19, CD2, CD5	Lymphoid antigen aberrancies
CD38, CD123, CD133	Leukemia stem cell identification

**Table 2:** Classification of AML according to ICC and WHO 2022.

ICC 2022		WHO 2022	
Category	Blasts Required for Diagnosis	Category	Blasts Required for Diagnosis
AML with Recurrent Genetic Abnormalities		AML with Defining Genetic Abnormalities	
Acute promyelocytic leukemia (APL) with t(15;17)(q24.1;q21.2)/*PML::RARA*	≥10%	Acute promyelocytic leukemia (APL) with *PML::RARA* fusion	No threshold
APL with other *RARA* rearrangements	≥10%		
AML with t(8;21)(q22;q22.1)/*RUNX1::RUNX1T1*	≥10%	AML with *RUNX1::RUNX1T1* fusion	No threshold
AML with inv(16)(p13.1q22) or t(16;16)(p13.1;q22)/*CBFB::MYH11*	≥10%	AML with *CBFB::MYH11* fusion	No threshold
AML with t(9;11)(p21.3;q23.3)/*MLLT3::KMT2A*	≥10%	AML with *KMT2A* rearrangement	No threshold
AML with other *KMT2A* rearrangements	≥10%		
AML with t(6;9)(p22.3;q34.1)/*DEK::NUP214*	≥10%	AML with *DEK::NUP214* fusion	No threshold
AML with inv(3)(q21.3q26.2) or t(3;3)(q21.3;q26.2)/*GATA2*; *MECOM(EVI1)*	≥10%	AML with *MECOM* rearrangement	No threshold
AML with other *MECOM* rearrangements	≥10%		
AML with other rare recurring translocations	≥10%	AML with *NUP98* rearrangement	No threshold
		AML with *RBM15::MRTFA* fusion	No threshold
AML with t(9;22)(q34.1;q11.2)/*BCR::ABL1*	≥20%	AML with *BCR::ABL1* fusion	≥20%
AML with mutated *NPM1*	≥10%	AML with *NPM1* mutation	No threshold
AML with in-frame bZIP *CEBPA* mutations	≥10%	AML with *CEBPA* mutation	≥20%
AML and MDS/AML with mutated *TP53*	10–19% (MDS/AML) and ≥20% (AML)		
AML and MDS/AML with myelodysplasia-related gene mutations Defined by mutations in *ASXL1*, *BCOR*, *EZH2*, *RUNX1*, *SF3B1*, *SRSF2*, *STAG2*, *U2AF1*, or *ZRSR2*	10–19% (MDS/AML) and ≥20% (AML)	AML, myelodysplasia-related Defined by one or more cytogenetics of molecular alterations: - Somatic mutations in *ASXL1*, *BCOR*, *EZH2*, *SF3B1*, *SRSF2*, *STAG2*, *U2AF1*, or *ZRSR2* - Cytogenetic abnormalities: complex karyotype (≥3 abnormalities); del(5q) or 5q loss; −7, del(7q) or 7q loss; del(11q); del(12p) or 12p loss; −13 or del(13q); del(17p) or 17p loss, i(17q), idic(X)(q13)	≥20%
AML with myelodysplasia-related cytogenetic abnormalities Defined by a complex karyotype (≥3 unrelated clonal chromosomal abnormalities in the absence of other class-defining recurring genetic abnormalities), del(5q)/t(5q)/add(5q), −7/del(7q), +8, del(12p)/t(12p)/add(12p), i(17q),−17/add(17p) or del(17p), del(20q), and/or idic(X)(q13) clonal abnormalities	10–19% (MDS/AML) and ≥20% (AML)		
		AML with other defined genetic alterations	No threshold
AML without recurrent genetic abnormalities		AML without defining genetic abnormalities	
AML not otherwise specified (NOS)	10–19% (MDS/AML) and ≥20% (AML)	AML defined by differentiation	≥20%

**Table 3:** Molecular AML markers and their clinical value.

Gene	Diagnostic	Prognostic	Therapeutic	MRD Marker
*FLT3*				
*IDH1*				
*IDH2*				
*NPM1*				
*CEBPA*				*
*DDX41*				*
*TP53*				*
*ASXL1*				
*BCOR*				
*EZH2*				
*RUNX1*				*
*SF3B1*				
*SRSF2*				
*STAG2*				
*U2AF1*				
*ZRSR2*				
*ANKRD26*				*
*BCORL1*				
*BRAF*				
*CBL*				
*CSF3R*				
*DNMT3A*				
*ETV6*				*
*GATA2*				*
*JAK2*				
*KIT*				
*KRAS*				
*NRAS*				
*NF1*				
*PHF6*				
*PPM1D*				
*PTPN11*				
*RAD21*				
*SETBP1*				
*TET2*				
*WT1*				
**Fusion Gene**	**Diagnostic**	**Prognostic**	**Therapeutic**	**MRD Marker**
*PML::RARA*				
*CBFB::MYH11*				
*RUNX1:RUNX1T1*				
*KMT2Ar*				
*BCR::ABL1*				
*DEK::NUP214*				
*MECOMr*				
*NUP98r*				
*KAT6A::CREBBP*				
*RBM15::MRTFA*				
*X::RARA*				

Grey colored cell: Marker with diagnostic, prognostic and/or therapeutic value in AML. Robust MRD marker. Less validated MRD marker. Potential MRD marker. * Excluded as MRD marker if suspicion of germline mutation (variant allele frequency higher than 30%). Excluded MRD marker.

## Data Availability

Data sharing not applicable.
